# Practical experience in commissioning ring applicators using ring applicator component type with bravos control software v1.2

**DOI:** 10.1002/acm2.70064

**Published:** 2025-05-02

**Authors:** Xiuxiu He, Michael A. Trager, Gil'ad N. Cohen, Antonio L. Damato, David Aramburu Núñez

**Affiliations:** ^1^ Department of Medical Physics Memorial Sloan Kettering Cancer Center New York USA; ^2^ Present address: Department of Radiation Medicine Center for Advanced Medicine Northwell Health New York USA; ^3^ Present address: Department of Radiation Oncology NYU Langone New York USA

**Keywords:** Commissioning, GYN intracavity brachytherapy, ring applicator

## Abstract

**Purpose:**

The Bravos v1.2 control software introduces a ring applicator component type (ACT) with channel length verification to address geometric characteristics and dosimetric discrepancies caused by source positioning inaccuracies. This study aims to commission the ring applicator and investigate the new ring ACT in Bravos 1.2.

**Material and methods:**

We evaluated two commissioning methods for a CT/MRI‐compatible titanium ring applicator across three Bravos afterloaders and compared the new ring ACT with the traditional rigid ACT. Modifications to Varian's standard commissioning method included: (1) Delivering film plans with a 0.5 cm step size instead of 1 cm; (2) Alternating 0.3 s and 1 s dwell positions for enhanced source positioning analysis; (3) Including both “odd” and “even” positions to replicate clinical conditions. Films for 30‐, 45‐, and 60‐degree rings (3.2 cm diameter) were delivered using modified methods and manual offsets of 0.0 cm, 0.1 cm, and 0.2 cm. Discrepancies between delivered and planned positions were analyzed, and the optimal offset was validated using clinical plans. Dosimetric differences for various gross tumor volumes (GTVs) and organs at risk (OARs) were assessed.

**Results:**

Film analysis (216 films) identified 0.2 cm as the optimal offset for all rings and afterloaders, minimizing deviations between the planned and delivered dwell positions. The rigid ACT showed larger discrepancies. The optimal offset reduced physical dosimetric differences to < 1% for key clinical metrics (D95, D90, D2cc) across all angles, with negligible differences in EQD2 values.

**Conclusion:**

A novel commissioning procedure was developed to determine an optimal offset for accurate source positioning and minimize dosimetric discrepancies with the ring ACT. This method improves accuracy compared to the rigid ACT and standardizes commissioning for Bravos afterloaders with the v1.2 control system.

## 1 INTRODUCTION

Brachytherapy, in association with external beam radiotherapy, is the gold standard for treating cervical cancer.^1^ Ring and tandem, tandem and ovoids, and hybrid applicators are the most common choices for clinicians when treating this disease, with high dose rate (HDR) brachytherapy being the most common treatment modality.^2^, ^3^, ^4^, ^5^, ^6^

The release of the Varian Bravos HDR afterloader in 2018, with its new feature for channel length verification, allows users to accurately determine the physical length of the applicator and transfer guide tube (TGT) combination prior to treatment. Bravos provides different applicator component type (ACT) settings depending on the type of applicator used for treatment, controlling dummy, and active source differently when different settings are selected. Its recently released control software (Version 1.2) introduced two new ACT, ring and needle, to rigid and flexible options. With each user‐specified ACT, the afterloader performs the automated channel length assessment using the dummy wire, verifying the total length of the channel. Varian Instructions For Use (IFU) indicate which ACT should be used for specific applicators, which takes into account specifics of the geometry, flexibility, and other characteristics of the applicators used.^7^ Ring ACT should be assigned to ring applicators during channel length verification and treatment. It is well known that ring applicators need to be more accurate in the dwell positions inside the ring. Uneven stepping of the source and slacking the active wire within the applicator's lumen could lead to more than 0.2 cm differences between planned and delivery positions.^8^, ^9^, ^10^, ^11^ This is attributed to the difference in source wire diameter compared to the inner diameter of the ring.

Technical notes and practical guidelines have been published with recommendations on how to commission ring applicators, verifying the accuracy of the source inside the ring applicator.^9^, ^11^ Nevertheless, the main challenge continues to be finding the correction offset that provides the more accurate planned versus delivered source position.^12^ It is recommended that individual commissioning of the combination of ring applicator and afterloader is performed due to variations among afterloaders and ring applicators. This work aims to assess the commissioning of a ring applicator with the new ring ACT introduced by Varian Bravos afterloader, its variability among Bravos afterloaders, and its clinical impact.

## 2 MATERIAL AND METHODS

### 2.1 Bravos afterloaders and applicators

Three Varian Bravos HDR remote afterloaders (Console software version 1.2) were used with titanium ring applicators (CT‐compatible/MR‐ conditional, Mick Radio‐Nuclear Instruments—an EZ Bebig company, Mt Vernon, NY) of a diameter of 3.2 cm and with ring angles of 30, 45, and 60 degrees. Compatible TGTs; were used. The total length of the TGT and the ring applicator is 150 cm. Varian position verification was performed using Varian's CamScale, and film position verification was performed as previously reported,^13^ before every batch of film exposure.

### 2.2 Varian method (VM)

To determine the optimal ring offset, the Varian method (VM) requires delivering ring dwell positions on film and comparing actual and planned dwell positions. The Varian procedure for ring applicator commissioning calls for using a 1 cm step size.^7^ This step size is larger than standard clinical practice but likely chosen to improve visualization of the dwell positions on the radiographs since 0.5 cm step size would cause the opaque film signal from adjacent dwell positions to be too close together and “bleed” into each other. Once films are delivered, users can first use the opaque film signal to identify the source dwell position and then determine the delivered position of each dwell by calculating the arc length. Note the ring radius as r and Δθ is the angle from the end of the ring's lumen to the center of the dwell position. Then, the arc length is r×Δθ, which is the delivered dwell position relative to the distal end of the lumen of the ring applicator.

The delivered position can then be compared with the planned position to determine their discrepancy. Once the discrepancy is known, the offset required to correct all positions can be determined.^7^ This method instructs users to deliver ring plans on film for “odd” positions. “Odd” positions start at the first, most distal dwell position, and we added “even” positions that start at the second‐most distal dwell position, with both variations continuing with every other position thereafter, assuming a 0.5 cm step size. This means delivering two sets of films with dwells 1 cm apart from each other starting at either the first (“odd”) or second (“even”) dwell position, as opposed to the typical 0.5 cm dwell spacing seen clinically.

### 2.3 Modified Varian method (MVM)

We adopted the VM for ring commissioning with slight modifications. To minimize the aforementioned opaque film signal “bleeding” into one another due to being too close together, the modification we implemented was to deliver film plans with a 0.5 cm step size instead of the suggested 1 cm to reproduce the clinical scenario. Note that we still separated the analysis into only “odd” or “even” dwells per film. To do so without hindering visualization of the dwell locations, we delivered alternating 0.3 s and 1 s dwell positions, where 0.3 s dwells were included for clinical accuracy in delivery without creating sufficient film opacity for reading, and 1 s dwell positions for “odd” or “even” analysis.

### 2.4 Plan generation and delivery

Two sets of plans were generated in the Bravos console for a nominal 10 Ci source following the VM and MVM guidelines. The first dwell position was set to 149.9 cm, leaving a 0.1 cm space between the channel end and the tip of the cable to ensure the source travels to the end of the applicator. For the VM plans, the source step size was set to be 1 cm, and plans were created for “even” or “odd” only dwell positions, such that the spacing between the planned dwell positions was maintained 1 cm apart. For the MVM plans, the source step size was set to be 0.5 cm, and plans were created delivering all dwell positions at 0.5 cm apart but alternating 0.3 s and 1 s dwells, with the first dwell position's time determined by “even” or “odd” delivery.

**FIGURE 1:**
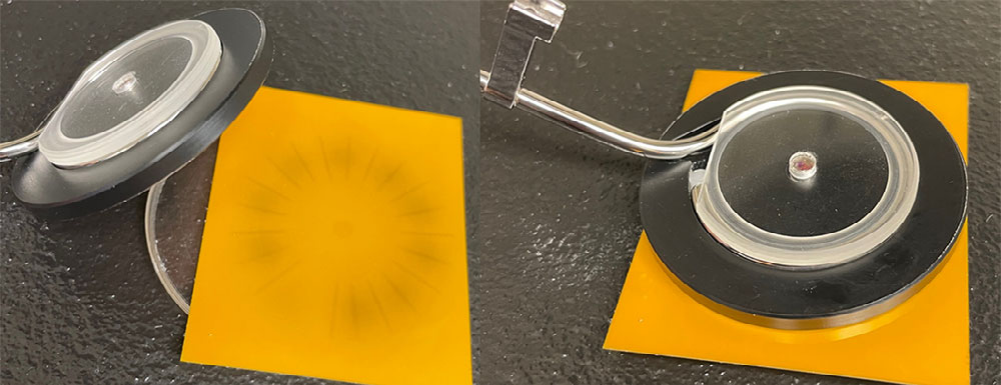
Autoradiograph acquisition setup. The film is fixed in a reproducible position using an in‐house phantom consisting of a groove for the ring applicator and a reproducible position concerning the tungsten markers for visualization of planned dwell positions.^14^

As mentioned earlier, the plans were delivered on the Bravos 1.2 afterloader for radiographic image acquisition using the ring or rigid ACT for 30, 45, and 60‐degree rings. During delivery, the ring applicator and TGT were stabilized, and the ring's surface was kept in close contact with the radiographic film (Gafchromic RTQA2 (Ashland, Bridgewater, NJ USA), pre‐cut to 5.5 cm × 5.5 cm squares), as shown in Figure 1. For delivered source position analysis, films are held with a novel phantom that consists of a groove to fix the ring applicator in relation to the embedded tungsten markers.^14^

The afterloader performed automatic channel length verification and applied automatic adjustments for length discrepancies of less than 0.2 cm. Analysis was performed by importing films to Varian's BrachyVision version 16.1 and following the VM of calculating arc length measurements based on the known ring diameter and angle between the end of the ring's lumen and the center of the delivered position.

Based on Varian IFU for ACT, all films for offset determination were delivered using the “ring” ACT as recommended by IFU. However, an additional set of films for all ring angles on one afterloader (afterloader 2) were delivered with the “rigid” ACT to determine if the “ring” ACT improves delivery accuracy.

### 2.5 Offset determination

To determine our clinic's ring offset for planning, we delivered films using a 0.5 cm step size for 30‐, 45‐, and 60‐degree rings with a diameter of 3.2 cm, and all three of our clinical afterloaders with 0 cm, 0.1 cm, and 0.2 cm offsets introduced while following our MVM approach. Discrepancies between delivered positions on film and planned dwell positions in BrachyVision (Varian® Medical Systems, Palo Alto, CA, USA) were determined. Average discrepancies between planned and delivered dwell positions were calculated for all dwells within the ring, as well as stratified by only those dwell positions typically used in our clinical cases: four lateral dwells on either side of the ring. The offset with the smallest discrepancy is chosen as the clinical offset required during planning.

### 2.6 Film import

Two hundred and sixteen films were delivered and analyzed: 108 for the VM and 108 for the MVM. Films were scanned using photo mode on an Epson Expression 12000XL with settings of reflective document type, 48‐bit color, 1200 dpi resolution, and high scanning quality. The scanned images were imported to Brachytherapy 2D Entry using a bitmap filter and then scaled to have the known radius of the ring's inner lumen. Subsequently, the scaled image was inserted as a new radiograph set with a single film geometry type and a magnification factor of 1.0.

### 2.7 Clinical dosimetric analysis

Three clinical cases were retrospectively selected for the 3.2 cm diameter ring at 30, 45, and 60‐degree angles to validate the optimal offset dosimetrically. Our clinical plans use a step size of 0.5 cm, adding an offset to the first dwell and moving the digitization of the inner lumen channel forward (proximal) to reproduce the actual delivered plan. The plans were executed and assessed based on historical points and organ volumes as recommended by the American Brachytherapy Society, the ICRU,^15^ and the physicians at MSKCC, such that the D90 to the cervix should be 100% of the prescription dose. The total D90 goal for the cervix, including external beam radiotherapy, is 85  Gy EQD2 or more. All plans were calculated in BrachyVision version 16.1 using the TG‐43 algorithm, which had no inhomogeneity correction and a calculation grid of 2.5 mm.

Besides the clinical GTVs, GTVs that are anterior and posterior ring/tandem intersection and within 1 cm superior to the cap were used to generate standard plans using the solid library applicators and then optimized such that the GTV for the plan is well covered. Thus, nine individual plans per ring angle for every offset were analyzed: 3 plans per GTV (planned, actual, and expected) and 3 GTVs (clinical, anterior, and posterior). To create comparison plans, we started with the original clinical loading patterns and dwell times for each patient, and then the dwell positions were shifted proximally, consistent with the applied overall offset. Three planned offsets, 0.0 cm, 0.1 cm, and 0.2 cm were applied to compare with the no‐offset clinical plans using dose calculated on the same CT volume and structure set.

The dosimetric differences between the clinical plans and those with the optimal offset applied were evaluated. For comparison, cervix (D95, D90), GTV (D100, D90), and the OAR (D2cc for bladder, rectum, bowel, and rectosigmoid) dosimetry were calculated in physical dose and EQD2. The percent and absolute differences were quantified by comparing with and without the optimal offset for physical dose and EQD2.

### 2.8 Measurement uncertainty

Uncertainty in measuring dwell positions on film was determined by repeating measurements from one set of “odd” and “even” films five times. All measurements in this study were performed by the same user to eliminate any inter‐user variability.

## 3 RESULTS

### 3.1 VM—ring ACT

**FIGURE 2:**
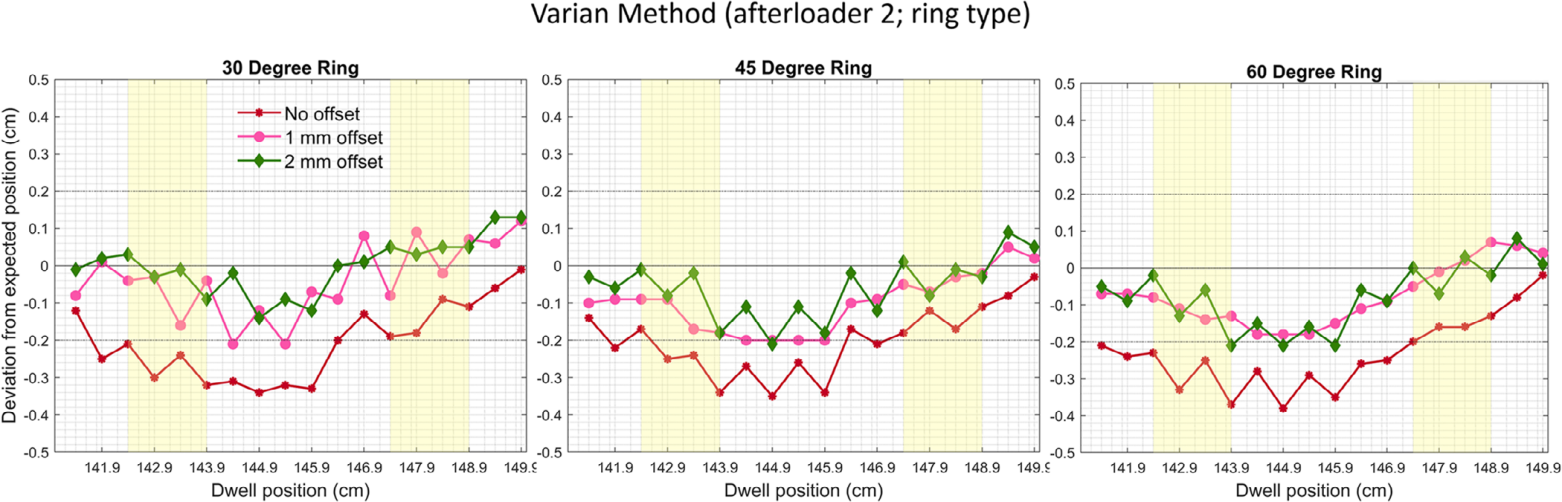
Deviations between planned and delivered dwell positions for 30‐, 45‐, and 60‐degree rings using the ring applicator component type. Designation with 0 cm, 0.1 cm, and 0.2 cm offsets was analyzed using the Varian method (VM) for offset commissioning. Areas highlighted in yellow are commonly used clinical dwell positions routinely used during ring and tandem treatments at our institution.

Graphical results for the analysis of the VM using the ring ACT designation can be seen in Figure 2. First dwell position deviations (displayed as 0/0.1/0.2 cm offsets) are 0.01 cm/‐0.12 cm/‐0.13 cm, 0.03 cm/‐0.02 cm/‐0.05 cm, and 0.02 cm/‐0.04 cm/‐0.01 cm for 30‐, 45‐, and 60‐degree rings, respectively. In addition, the deviations for the second dwell positions for 0/0.1/0.2 cm offsets are 0.06 cm/‐0.06 cm/‐0.13 cm, 0.08 cm/‐0.05 cm/‐0.09 cm, and 0.08 cm/‐0.06 cm/‐0.08 cm for 30‐, 45‐, and 60‐degree rings.

For the 30‐degree ring, average absolute deviations throughout the ring between planned and delivered positions were 0.21 ± 0.10 cm, 0.09 ± 0.06 cm, and 0.06 ± 0.05 cm for 0 cm, 0.1 cm, and 0.2 cm offsets, respectively. For the 45‐degree ring, average absolute deviations throughout the ring between planned and delivered positions were 0.20 ± 0.09 cm, 0.11 ± 0.07 cm, and 0.08 ± 0.06 cm for 0 cm, 0.1 cm, and 0.2 cm offsets, respectively. For the 60‐degree ring, average absolute deviations throughout the ring between planned and delivered positions were 0.23 ± 0.10 cm, 0.10 ± 0.05 cm, and 0.09 ± 0.07 cm for 0 cm, 0.1 cm, and 0.2 cm offsets, respectively.

For the 30‐degree ring, average absolute deviations stratified by commonly used clinical ring positions between planned and delivered positions were 0.23 ± 0.09 cm, 0.09 ± 0.06 cm, and 0.05 ± 0.05 cm for 0 cm, 0.1 cm, and 0.2 cm offsets, respectively. For the 45‐degree ring, average absolute deviations stratified by commonly used clinical ring positions between planned and delivered positions were 0.23 ± 0.08 cm, 0.12 ± 0.07 cm, and 0.08 ± 0.07 cm for 0 cm, 0.1 cm, and 0.2 cm offsets, respectively. For the 60‐degree ring, average absolute deviations stratified by commonly used clinical ring positions between planned and delivered positions were 0.26 ± 0.08 cm, 0.11 ± 0.06 cm, and 0.10 ± 0.08 cm for 0 cm, 0.1 cm, and 0.2 cm offsets, respectively.

### 3.2 MVM—ring ACT

**FIGURE 3:**
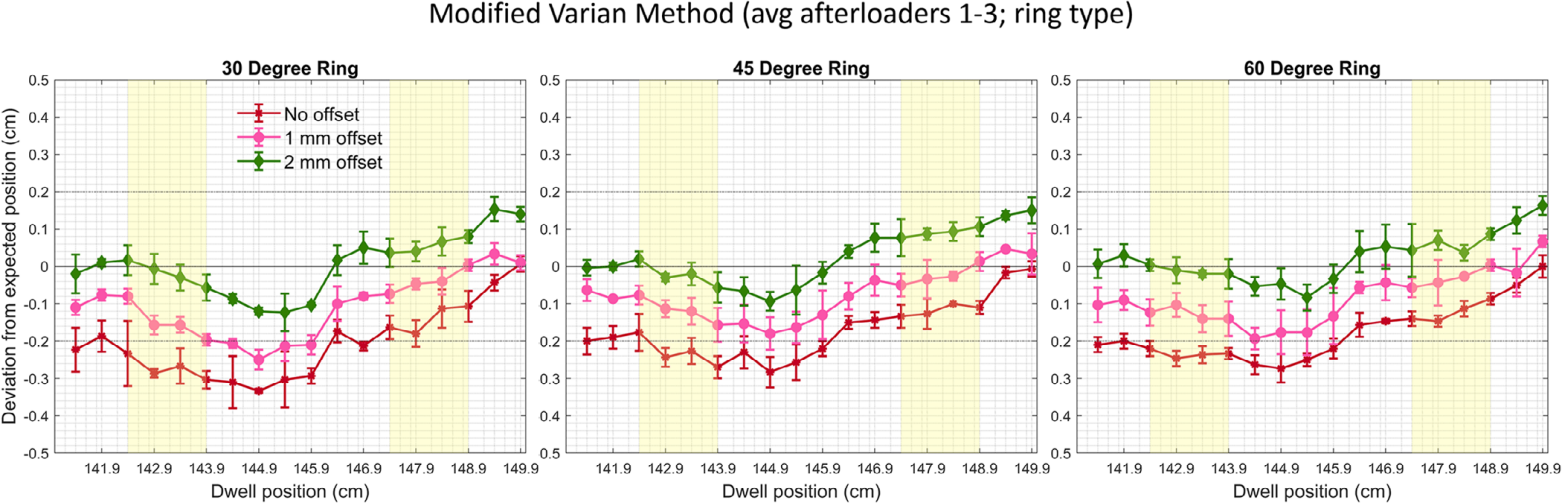
Deviations between planned and delivered dwell positions for 30‐, 45‐, and 60‐degree rings using the ring applicator component type averaged over three afterloaders. Designation with 0 cm, 0.1 cm, and 0.2 cm offsets was analyzed using the modified Varian method (MVM) for offset commissioning. Areas highlighted in yellow are commonly used clinical dwell positions routinely used during ring and tandem treatments at our institution.

Average results across all afterloaders and dwell positions for the analysis of the MVM using the ring ACT designation can be seen in Table 1. Graphical results for average deviations across all afterloaders for all individual dwell positions data are in Figure 3. Error bars represent the standard deviation between each afterloader's results. The deviation between the first delivered and planned dwell position increases with the increasing introduced offset. First dwell position deviations (displayed as 0/0.1/0.2 cm offsets) are ‐0.01 ± 0.02 cm/‐0.01 ± 0.02 cm/0.14 ± 0.02 cm, 0.01 ± 0.02 cm/‐0.03 ± 0.06 cm/‐0.15 ± 0.03 cm and 0.00 ± 0.03 cm/‐0.07 ± 0.02 cm/‐0.16 ± 0.03 cm for 30‐, 45‐, and 60‐degree rings, respectively. In addition, the deviations for the second dwell positions for 0/0.1/0.2 cm offsets are 0.04 ± 0.02 cm/‐0.03 ± 0.02 cm/0.15 ± 0.03 cm, 0.02 ± 0.02 cm/‐0.05 ± 0.01 cm/‐0.14 ± 0.01 cm and 0.05 ± 0.02 cm/0.02 ± 0.06 cm/‐0.12 ± 0.04 cm for 30‐, 45‐, and 60‐degree rings.

**TABLE 1:** Average deviations between planned and delivered dwell positions on afterloaders 1, 2, and 3 for 30‐, 45‐, and 60‐degree rings using the ring applicator component type (ACT) with 0 cm, 0.1 cm, and 0.2 cm offsets analyzed using the modified Varian method (MVM) for offset commissioning stratified by all dwell positions or commonly used clinical ring dwell positions (designated “TnR”). Uncertainty values indicated are the propagated error from standard deviations between all afterloaders for different offsets and ring angles and standard deviations amongst data sets for a given afterloader and ring combination.

	Average deviations from planned positions for afterloaders 1–3 using the modified Varian method and ring applicator component type (cm)								
	30 Degree ring			45 Degree ring			60 Degree ring		
**Offset**	**0** **cm**	**0.1** **cm**	**0.2** **cm**	**0** **cm**	**0.1** **cm**	**0.2** **cm**	**0** **cm**	**0.1** **cm**	**0.2** **cm**
**Avg**	0.21 ± 0.1	0.11 ± 0.08	0 ± 0.08	0.17 ± 0.08	0.08 ± 0.07	−0.02 ± 0.07	0.18 ± 0.08	0.09 ± 0.07	−0.02 ± 0.06
**ABS avg**	0.21 ± 0.09	0.11 ± 0.08	0.06 ± 0.05	0.17 ± 0.08	0.09 ± 0.05	0.06 ± 0.04	0.18 ± 0.08	0.09 ± 0.06	0.05 ± 0.04
**Avg (TnR)**	0.19 ± 0.11	0.11 ± 0.10	−0.01 ± 0.10	0.16 ± 0.09	0.07 ± 0.08	−0.04 ± 0.08	0.16 ± 0.08	0.08 ± 0.08	−0.03 ± 0.07
**ABSavg (TnR)**	0.23 ± 0.08	0.13 ± 0.08	0.06 ± 0.04	0.19 ± 0.06	0.10 ± 0.06	0.06 ± 0.03	0.20 ± 0.06	0.10 ± 0.06	0.04 ± 0.03

For the 30‐degree ring, average absolute deviations throughout the ring between planned and delivered positions were 0.21 ± 0.09 cm, 0.11 ± 0.07 cm, and 0.06 ± 0.05 cm for 0 cm, 0.1 cm, and 0.2 cm offsets, respectively. For the 45‐degree ring, average absolute deviations throughout the ring between planned and delivered positions were 0.17 ± 0.08 cm, 0.09 ± 0.05 cm, and 0.06 ± 0.04 cm for 0 cm, 0.1 cm, and 0.2 cm offsets, respectively. For the 60‐degree ring, average absolute deviations throughout the ring between planned and delivered positions were 0.18 ± 0.08 cm, 0.09 ± 0.06 cm, and 0.05 ± 0.04 cm for 0 cm, 0.1 cm, and 0.2 cm offsets, respectively.

For the 30‐degree ring, average absolute deviations stratified by commonly used clinical ring positions between planned and delivered positions were 0.23 ± 0.08 cm, 0.13 ± 0.08 cm, and 0.06 ± 0.04 cm for 0 cm, 0.1 cm, and 0.2 cm offsets, respectively. For the 45‐degree ring, average absolute deviations throughout the ring between planned and delivered positions were 0.19 ± 0.06 cm, 0.10 ± 0.06 cm, and 0.06 ± 0.03 cm for 0 cm, 0.1 cm, and 0.2 cm offsets, respectively. For the 60‐degree ring, average absolute deviations throughout the ring between planned and delivered positions were 0.20 ± 0.06 cm, 0.10 ± 0.06 cm, and 0.04 ± 0.03 cm for 0 cm, 0.1 cm, and 0.2 cm offsets, respectively.

### 3.3 MVM—rigid ACT

**FIGURE 4:**
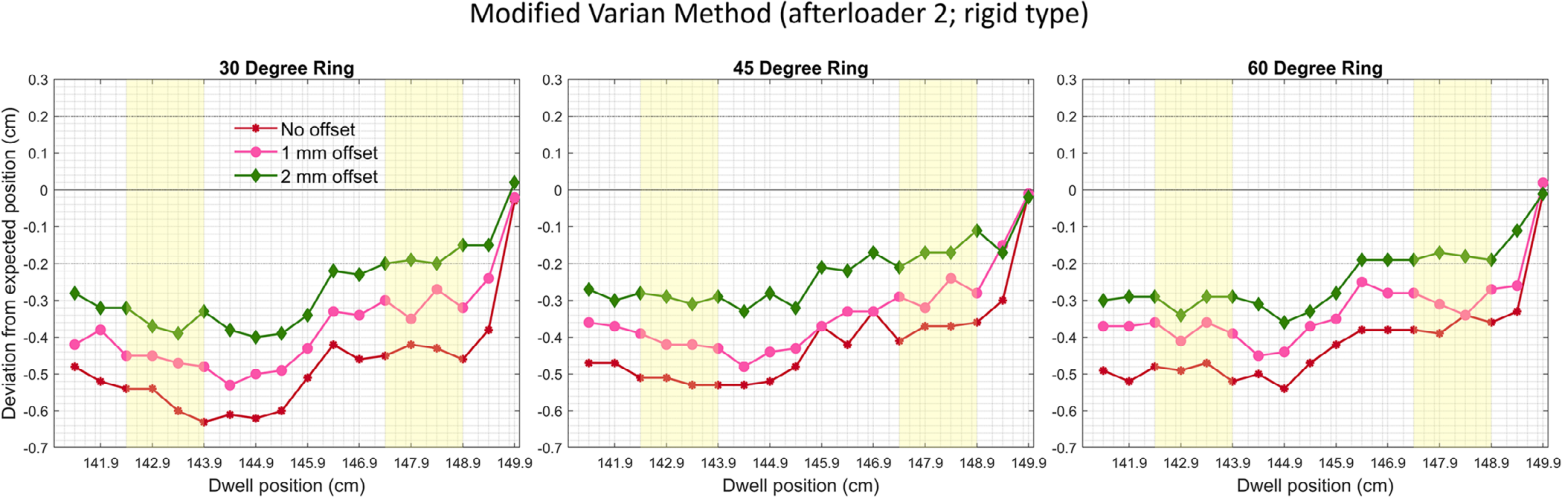
Deviations between planned and delivered dwell positions for 30‐, 45‐, and 60‐degree rings using the rigid applicator component type. Designation with 0 cm, 0.1 cm, and 0.2 cm offsets was analyzed using the modified Varian method (MVM) for offset commissioning. Areas highlighted in yellow are commonly used clinical dwell positions routinely used during ring and tandem treatments at our institution.

Figure 4 shows graphic results for the analysis of the MVM using the rigid ACT designation. The first delivered dwell position is within 0.03 cm of planned positions for 30‐, 45‐, and 60‐degree rings with all offsets.

**FIGURE 5:**
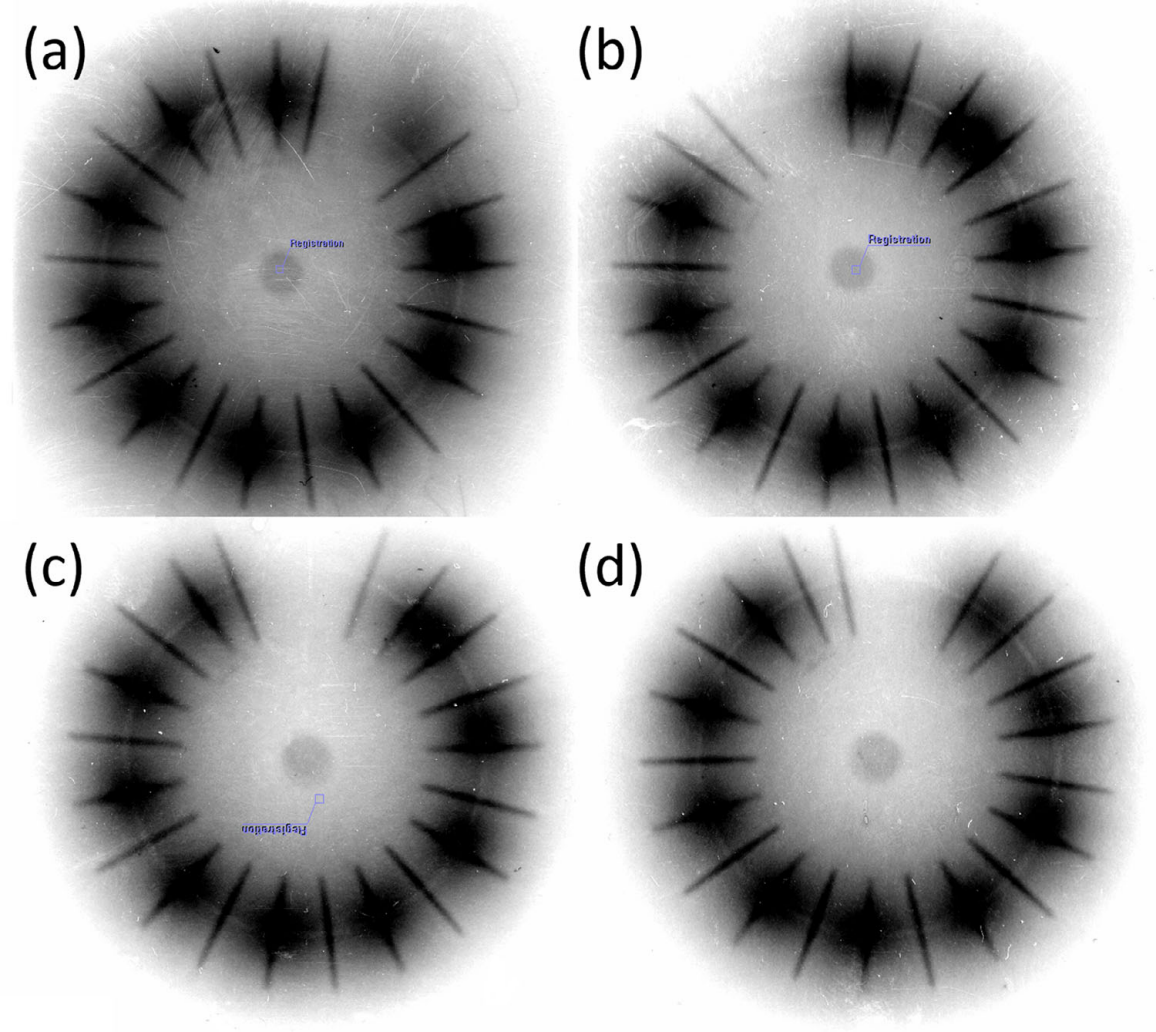
Examples of radiograph using Varian Method ((a) and (b)) and Modified Varian Method ((c) and (d)). (a) and (c) show the odd dwell positions. (b) and (d) show the even dwell positions.

For the 30‐degree ring, average absolute deviations throughout the ring between planned and delivered positions were 0.48 ± 0.14 cm, 0.38 ± 0.12 cm, and 0.27 ± 0.11 cm for 0 cm, 0.1 cm, and 0.2 cm offsets, respectively. For the 45‐degree ring, average absolute deviations throughout the ring between planned and delivered positions were 0.42 ± 0.13 cm, 0.34 ± 0.11 cm, and 0.23 ± 0.08 cm for 0 cm, 0.1 cm, and 0.2 cm offsets, respectively. For the 60‐degree ring, average absolute deviations throughout the ring between planned and delivered positions were 0.42 ± 0.12 cm, 0.33 ± 0.10 cm, and 0.24 ± 0.09 cm for 0 cm, 0.1 cm, and 0.2 cm offsets, respectively (Figure 4). Examples of radiograph using the VM and MVM are shown in Figure 5.

For the 30‐degree ring, average absolute deviations stratified by commonly used clinical ring positions between planned and delivered positions were 0.52 ± 0.08 cm, 0.41 ± 0.09 cm, and 0.29 ± 0.09 cm for 0 cm, 0.1 cm, and 0.2 cm offsets, respectively. For the 45‐degree ring, average absolute deviations stratified by commonly used clinical ring positions between planned and delivered positions were 0.45 ± 0.08  cm, 0.37 ± 0.08 cm, and 0.24 ± 0.07 cm for 0 cm, 0.1 cm, and 0.2 cm offsets, respectively. For the 60‐degree ring, average absolute deviations stratified by commonly used clinical ring positions between planned and delivered positions were 0.44 ± 0.07 cm, 0.35 ± 0.05 cm, and 0.26 ± 0.07 cm for 0 cm, 0.1 cm, and 0.2 cm offsets, respectively.

### 3.4 Offset determination

The optimal offset for all rings and afterloaders was decided based on results from the film analysis using the MVM presented. Optimal offset results in the smallest deviation between planned and delivered positions. The offset resulting in the best agreement between planned and delivered positions for afterloaders 1 and 2 was 0.2 cm for all ring angles and when considering all ring positions or stratifying by only commonly used clinical ring positions. The offset resulting in the best agreement between planned and delivered positions for afterloader 3 was also 0.2 cm across the board, with one exception. For a 60‐degree ring with afterloader 3, a 0.1 cm offset produced better agreement when considering all ring positions, and a 0.2 cm offset produced better agreement when stratified by commonly used clinical ring positions. However, for 0.1 cm and 0.2 cm offsets, the average deviations for all positions and those stratified by commonly used clinical ring positions were within measurement uncertainty. Among all the afterloaders and ring angles, the 0.2 cm offset has the optimal agreement between planned and delivered positions.

Analysis of the VM films also resulted in a 0.2 cm offset, providing the smallest deviation between planned and delivered dwell positions. The VM, however, due to the 1 cm step size, provided data that may not accurately represent the clinical delivery scenario. As shown in Figure 2, the difference in the deviation between individual planned and delivered dwell positions is bigger than that between even and odd dwell positions. Quantitatively, in the VM method, for the afterloader 2 and ring ACT, the max difference between neighboring dwell positions can have a magnitude of 0.16 cm, while the max difference for MVM is 0.1 cm.

Dwell position deviation results for all 0.2 cm offset data using the MVM with ring ACT designation on Bravos (averages of afterloaders 1, 2, and 3), VM with ring ACT on Bravos (afterloader 2), and MVM with rigid ACT on Bravos (afterloader 2) are presented in Table 2.

**TABLE 2:** Percentage difference of D90% and D95% for cervix, and D100% for GTV, and D2cc for Bladder, Rectal Sigmoid, Rectum, and Bowel between the clinical plan and the recreated plan with 0.1 cm or 0.2 cm offset for 3.2 cm diameter rings of angle 30, 45, and 60 degrees.

	30 Degree ring			45 Degree ring			60 Degree ring		
Offset	0.0 cm	0.1 cm	0.2 cm	0.0 cm	0.1 cm	0.2 cm	0.0 cm	0.1 cm	0.2 cm
**D95_HRCTV_Clinical**	0.6%	0.1%	0.6%	−0.3%	−0.1%	0.1%	**1.0%**	0.5%	0.1%
**D90_HRCTV_Clinical**	0.3%	0.2%	0.4%	−0.3%	−0.1%	0.1%	0.4%	0.1%	0.0%
**D100_GTV_Clinical**	−0.9%	−**1.5%**	−**1.0%**	0.6%	−0.4%	0.3%	−***3.0%***	−***2.9%***	−0.7%
**D90_GTV_Clinical**	−1.0%	0.7%	−**1.1%**	−0.1%	0.4%	−0.3%	−***2.3%***	−***2.5%***	−0.3%
**D2cc_Bladder**	0.3%	0.1%	0.0%	0.7%	0.8%	−0.4%	−***3.1%***	−***3.6%***	−0.8%
**D2cc_Rectum**	0.4%	0.0%	0.0%	0.1%	−0.4%	0.3%	***2.8%***	**1.4%**	0.6%
**D2cc_Bowel**	0.1%	0.0%	0.2%	0.0%	0.0%	0.0%	−0.8%	−0.8%	−0.4%
**D2cc_Rectosigmoid**	−0.1%	−0.1%	0.8%	−0.5%	−0.4%	0.3%	***2.7%***	***2.2%***	**1.0%**
**D95_HRCTV_Ant**	0.5%	0.1%	0.0%	−0.4%	−0.2%	0.2%	−0.2%	−0.2%	0.2%
**D90_HRCTV_Ant**	0.3%	0.1%	0.0%	−0.3%	−0.2%	0.1%	0.0%	0.0%	0.1%
**D100_GTV_Ant**	0.7%	0.2%	−0.2%	−0.2%	0.2%	−0.2%	**2.0%**	0.9%	−0.4%
**D90_GTV_Ant**	0.4%	0.2%	−0.2%	0.4%	0.6%	−0.4%	0.1%	0.4%	−0.1%
**D2cc_Bladder**	0.4%	0.2%	−0.2%	0.6%	1.0%	−0.6%	***2.4%***	**1.3%**	−0.8%
**D2cc_Rectum**	0.3%	−0.2%	0.6%	0.2%	−0.5%	0.4%	0.9%	−0.2%	0.1%
**D2cc_Bowel**	0.1%	0.0%	0.0%	0.0%	−0.1%	0.1%	0.6%	0.3%	−0.5%
**D2cc_Rectosigmoid**	−0.1%	−0.1%	0.1%	−0.5%	−0.4%	0.3%	−0.7%	−0.3%	0.6%
**D95_HRCTV_Pst**	**1.1%**	0.8%	−0.2%	0.2%	0.4%	−0.2%	0.0%	−0.1%	0.1%
**D90_HRCTV_Pst**	0.7%	0.7%	−0.1%	0.0%	0.1%	−0.1%	−0.2%	0.0%	0.0%
**D100_GTV_Post**	−0.3%	0.4%	−0.1%	−**1.9%**	−**1.3%**	0.5%	**1.6%**	−0.5%	0.2%
**D90_GTV_Post**	0.0%	0.3%	0.0%	−0.7%	−0.7%	0.4%	***3.0%***	−0.5%	0.1%
**D2cc_Bladder**	0.3%	0.6%	−0.1%	0.8%	0.9%	−0.5%	−***3.5%***	**1.3%**	−0.9%
**D2cc_Rectum**	−0.2%	−0.1%	0.9%	−0.4%	−0.7%	0.5%	***4.4%***	−0.1%	−0.1%
**D2cc_Bowel**	0.1%	0.3%	−0.1%	0.0%	−0.1%	0.1%	−**1.1%**	0.3%	−0.5%
**D2cc_Rectosigmoid**	−0.1%	0.2%	0.2%	−1.0%	−0.7%	0.5%	***4.7%***	−0.1%	0.2%

### 3.5 Measurement uncertainty

The average of standard deviations amongst five measurements for all ring dwell positions was 0.018 cm (Range: 0.004–0.031). The absolute mean deviation from the mean amongst five measurements for all ring dwell positions was 0.015 cm (Range: 0.003–0.027). The average standard deviation from the mean amongst five measurements for all ring dwell positions was 0.000 cm (Range: 0.000–0.001).

### 3.6 Clinical dosimetry

Clinical plans were randomly selected retrospectively for 3.2 cm ring angles of 30, 45, and 60 degrees for clinical dosimetric evaluation. Delivery recreated plans with offsets of 0.0 cm, 0.1 cm, and 0.2 cm were evaluated for the ring ACT. The percentage physical dose differences are shown in Table 2.

Compared with no offset, the 0.2 cm offset reduces the average dosimetric discrepancy between the planned and delivered. The physical dosimetric difference is less than 1% for D95 and D90 cervix, D2cc bladder, rectum, bowel, and rectosigmoid for all ring angles, while EQD2 is less than 0.11 for all ring angles. The dosimetric difference for cervix D95, D90, and OAR D2cc by 0.1% in the physical dose and EQD2 by 0.11, 0.11, and 0.09 Gy for the 30, 45, and 60‐degree rings, respectively.

## 4 DISCUSSION

For commissioning ring applicators with our three Bravos afterloaders, we sought to determine a single offset to best fit all afterloaders and ring angles. While doing so, we adopted the VM for ring commissioning with slight modifications to reproduce an actual clinical scenario better. We use the 0.5 cm step size at our clinic. We delivered alternating 0.3 s and 1 s dwell positions, where 0.3 s dwells were meant to ensure movement of the source was consistent with clinical delivery but not long enough to create a meaningful signal on film, and 1 s dwells to allow for adequate signal in determining dwell position location analysis. Therefore, in analyzing both “odd” and “even” films with this method, we mimic a clinical delivery of dwell positions and can visualize dwells accurately. VM modified method removes inconsistencies between odd and even exposures compared to the VM method, reducing uncertainties and making the commissioning process closer to clinical delivery conditions when determining the offset. These highlighted dwells correspond to four clinically commonly used lateral positions on either side of the ring. One set of films strictly following the VM was taken to determine if the MVM approach is necessary for an accurate offset or if delivered with a 1 cm step size is sufficient. Our results for rigid ACT are consistent with prior published results for Bravos V1.1.^16^ Following the trend in Figure 2, we can assume that a 0.4 cm offset would likely result in the best results for ACT rigid. For our clinical practice, a 0.4 cm offset will not be applicable as the dwell positions can be shifted dramatically, given the step size of 0.5 cm. Even though this is a projection, results for 0.0, 0.1, and 0.2 cm show a consistent trend. All films for offset determination were done using the “ring” ACT as recommended by the IFU. However, an additional set of films for all ring angles on one afterloader (afterloader 2) were delivered with the “rigid” ACT to determine if the “ring” ACT improves delivery. Our results show a high standard deviation on VM when compared to MVM, as shown in Figures 3 and 4, and positive deviations indicate actual dwell positions that are more distal to the end of the ring's lumen than the planned (expected) position and negative deviations indicate actual dwell positions that are more proximal than the planned position. The dwell positions have fewer smooth transitions in VM, which are unrealistic compared to clinical plans and, therefore, harder to conclude the optimized offset. Prior studies investigated the attribution of the evolution of the physical properties of the wire due to repeated deployments showing no variability.^16^ However, dummy and active wire, and applicator QA is paramount for ensuring accuracy of treatment delivery.

The optimal offset of this study was quantified to minimize the discrepancy between the planned and delivered ring dwell positions. Channel length measurement assessment is performed by Bravos before every delivery by measuring total channel length, ensuring accurate delivery. The automatic adjustment is inherent to the delivery and dependent on the applicator, TGT, and active and dummy wires used. Applicators and TGTs QA, along with a robust daily QA program is recommended to minimize the variability of the length adjustments. All automatic channel length adjustments by Bravos performed with either ring or rigid ACT were consistent for the different ring angles commissioned. Moreover, Failure modes and effects analysis can follow the guidance detailed in AAPM's TG‐100 report or refer to published examples^17^, ^18^, ^19^ by evaluating the likelihood of occurrence, the severity of the effect if the failure mode is not caught, and the lack of detectability.

The VM for determining a single ring offset value at applicator commissioning corrects for the discrepancy between planned and delivered ring dwell positions.^7^ During planning, the offset value is then implemented by intentionally over‐digitizing the ring by the determined offset value and adding this same value to the first source position of the channel. This essentially moves the first dwell position back to the correct location at the distal end of the ring's lumen in the plan while implementing the determined offset and maximizing agreement between planned and delivered positions, as shown in the clinical validation. Choosing an optimal offset is best across all afterloaders and angles, making planning straightforward and least error‐prone.^9^, ^20^

## 5 CONCLUSION

We developed a ring applicator commissioning procedure to derive optimal offset for accurate source positioning and minimize clinical dosimetric differences. We observed improvement in positioning inaccuracies comparing the ring versus rigid applicator component type. Clinical commissioning of ring applicators using MVM provides a better assessment of the offset needed for correcting plan and delivery positions, especially when a 0.5 cm step size is used clinically; MVM is an alternative commissioning strategy in ring applicator commissioning for the Bravos afterloader with the v1.2 control system.

## AUTHOR CONTRIBUTIONS

The authors confirm their contribution to the study as follows: study conception and design: Gil'ad N. Cohen, David Aramburu Núñez, Michael A. Trager, and Xiuxiu He; data acquisition and analysis: Xiuxiu He, Michael A. Trager, David Aramburu Núñez, and Gil'ad N. Cohen; interpretation of results: Xiuxiu He, Michael A. Trager, David Aramburu Núñez, Gil'ad N. Cohen, and Antonio L. Damato; draft manuscript preparation: Xiuxiu He, David Aramburu Núñez, Michael A. Trager, Gil'ad N. Cohen, and Antonio L. Damato; All the authors contributed significantly to the performed work, reviewed the results, and approved its final version to be published.

## CONFLICT OF INTEREST STATEMENT

The authors declare no conflicts of interest.

## Data Availability

Data available on request from the corresponding author.
